# Interdisciplinary Perspectives on Maintenance of Health Behavior Change

**DOI:** 10.1093/geroni/igaa057.2976

**Published:** 2020-12-16

**Authors:** Jaime Hughes, Janet Bettger, Susan Hughes, Mina Raj

**Affiliations:** 1 Duke University School of Medicine, Durham, North Carolina, United States; 2 University of Illinois at Chicago, Chicago, Illinois, United States; 3 University of Michigan, Ann Arbor, Michigan, United States

## Abstract

Modifying health behaviors can be difficult, especially for older adults who are challenged by multiple chronic conditions, reduced functional and/or cognitive capacity, and limited social support. Although much attention has been given to the theories, skills, and resources behind initiating and achieving behavior change, less work has focused on maintenance of health behaviors over time. This presentation will showcase pilot research inspired by RCCN’s first workshop, Achieving and Sustaining Behavior Change. Specifically, this pilot brings together an interdisciplinary team of behavioral scientists and health services researchers working at the intersection of intervention science and implementation science to better understand the construct of maintenance and discuss emerging methods for intervention development and evaluation. The presentation will utilize physical activity as an example behavior to demonstrate the value of interdisciplinary research, including recommendations on how some of the six NIA research centers can make unique contributions to understanding health behavior maintenance.

